# Impact of prenatal famine exposure on adulthood fasting blood glucose level

**DOI:** 10.1038/s41598-022-10120-3

**Published:** 2022-04-13

**Authors:** Kalkidan Hassen Abate, Getachew Arage, Habtamu Hassen, Jemal Abafita, Tefera Belachew

**Affiliations:** 1 Food and Nutrition Research Institute, Jimma University, Jimma, Ethiopia; 2 Department of Nutrition and Dietetics, College of Health Sciences, DebreTabor University, Debre Tabor, Ethiopia; 3 Department of Public Health, Hosanna College of Health Science, Hosanna, Ethiopia; 4 College of Business and Economics, Jimma University, Jimma, Ethiopia

**Keywords:** Diseases, Endocrinology, Risk factors

## Abstract

In the past decade, in low-income countries, there have been a rapid rise in prevalence of diabetes among adult population. Hence, understanding the context specific drivers of this change including the impacts of childhood nutrition adversaries on adult metabolic conditions is critical undertaking. This study investigates the potential effects of prenatal famine exposure to the Ethiopian great famine (1983–1985) on adulthood blood glucose level of survivors. A total of 441 adults (222 exposed and 219 controls) were included in the study. Self-reported place of birth and, date of birth and/or age were used to identify participants. A multivariable linear regression analysis was used to analyze the impact of prenatal famine exposure on the level of fasting blood glucose. In linear regression, unadjusted model (Model 1), fasting blood glucose level was increased by 4.13 (β = 4.13; 95% CI .41, 7.42) points in prenatal famine exposed groups, compared with non-exposed. Similarly, the positive association of prenatal famine exposure and fasting blood glucose level was maintained after adjusted for sex (Model 2) (β = . 4.08 95% CI .056, 7.50). Further adjusted for age, residence, educational status, wealth index and family size (Model 3) resulted in 4.10 (β = . 4.10 95% CI .45, 7.56) points increases in fasting blood glucose level. In model 4 adjusting for dietary pattern, physical activity level and family history of diabetes, alcohol and cigarette smoking resulted in 3.90 (β = 3.90, 95% CI 039, 7.52) points increase in fasting glucose level. In the he full adjusted model (Model 5) prenatal exposure to famine was resulted in 3.78 (β = 3.78, 95% CI .22, 7.34) increases in fasting blood glucose level after adjusted for BMI and waist to height ratio. There existed a positive association of prenatal famine exposure and adulthood blood glucose levels. In this population, establishing effective overweight/obesity prevention programs to minimize the co-impact of early famine exposure on blood glucose control are important.

## Introduction

Suboptimal blood glucose control manifested as hyperglycemia, or diabetes is the major concern of the healthcare system across the globe^1^. According to the world health organization, in 2019, an estimated 1.5 million global deaths were directly caused by diabetes^2^. Sub-Saharan Africa (SSA) countries are expected to have the fastest increase in the number of people living with diabetes in the next two decades^3^. In 2019, estimates of the Institute for Health Metrics and Evaluation (IHME) indicated that 2.35% (1.97–2.64) of the total death in Ethiopia was attributable to diabetes^4^.

The established risk factors for development of suboptimal blood glucose control in adults includes obesity, physical inactivity, unhealthy diet, increased blood pressure and genetic susceptibility^4,5^. On the other hand, the developmental origins of health and disease (DOHaD) hypothesis suggested that risk factors for suboptimal blood glucose control could have been linked to adaptive responses employed in early life following health and nutrition adversaries^6^. Nutritional deprivation in critical periods of growth and development, particularly in the first 1000 days, exert disproportionate influence on body organs to have short-term survival, but could have long term health consequences^7,8^.

Historical famines including “the Ethiopian great famine (1983–1985)” can be used as a natural experimental study framework where ‘famine’ can be taken as proxy exposure for ‘nutritional deprivation’ to investigate the short- and long-term metabolic consequences^9–11^. The Ethiopian great famine of 1983–85 was one of Africa’s most severe famines that caused over half a million deaths^12^. It affects the whole country, while the epicenter was in the northern provinces of Tigray and Wollo^13^. It was ranked as “Great” famine in most scales due to its highest casualty as compared to other famines in Ethiopia or elsewhere^12,13^.

Previous famine studies conducted in Asia, Europe, and Africa contributed a substantial body of evidence on the association of prenatal exposure to famine and hyperglycemia and or development of diabetes mellitus in adulthood^14–30^. However, these reports indicated inconsistent findings where the majority reported higher risk of hyperglycemia and or diabetes with the exposure^19–28^. Conversely, there existed studies which revealed null findings^14–18^ and an inverse association of early childhood famine exposure and impaired glucose metabolism during adulthood^19^. Furthermore, studies also documented that the association between early-life exposure to famine and the risk of hyperglycemia in adulthood appears to be moderated by covariates such as obesity, wealth, sex, and post famine environment later in life^19,20,22–24^.

In the present study, we have investigated the association of parental prenatal exposure to the Ethiopian great famine (1983–1985) with adulthood blood glucose levels in Wollo province, Ethiopia.

## Methods

### Participants and selection

This study is conducted in Raya Kobo district of Wollo province Northeast part of Ethiopia, which was the epicenter of the 1983–1985 Ethiopian great famine^12^. The exposed and control group has been described elsewhere in detail^31–34^. Briefly, participants were recruited on the basis of self-reported place of birth, birth date and/or age related to the famine period (Aug 1983 to Aug 1985)^12^. Accordingly, the study participants were categorized into two groups; prenatal exposed (age = 34 to 36 years; birth date = August 8, 1983 to August 30, 1985) and non-exposed groups (age** = **30 to 32 years**;** birth date = September 8, 1987 to October 8, 1988). Participants born immediately after end of the famine were excluded to account for transition period between exposed and non-exposed group. Thus, participants born between 8, September 1986 to 30, August 1987 were excluded from the study in order to minimize misclassification of famine exposure.

Multistage stratified random sampling technique was used to recruit our sample. First the baseline survey was conducted on selected Kebeles to identify eligible participants and a sampling frame was prepared. Finally, study participants were selected from each Kebeles using simple random sampling method from the prepared sampling frame. The flow diagram of the sampling method was shown in Supplementary file (Fig. 1).

Two population proportion formula using Epi-Info, taking type one error 5%, 80% power, design effect of 1.5, 5% non-response rate and a 1:1 ratio of exposed group to non-exposed group (r = 1) was used to calculate the total sample size. Assuming the prevalence of diabetes mellitus in prenatal exposed group (22.6%) and non-exposed group (9.8%) from a study conducted in China^25^, a total of 456 (228 exposed and 228 non-exposed) participate were selected. A total of 441 (222 exposed and 219 non-exposed groups) participants who had a complete data on all variables were included in the final analysis. Adults who were displaced to other regions of the country and those who were born in other locations during the famine years, physically disabled subjects with deformity (Kyphosis, Scoliosis, and limb deformity), participants diagnosed with diabetes, and pregnant women were excluded from the study.

### Measurements

The data were collected in accordance of the World Health Organization STEPwise approach to non-communicable disease risk-factor surveillance guideline^35^. Permission to conduct the study was obtained from the Institutional Review Board of Jimma University, Institute of Health Sciences, Ethiopia in accordance with guidelines of the Declaration of Helsinki (reference no. JHRPGD/660/2019). Participants background characteristics such as sex, age, smoking, alcohol drinking, physical activity, wealth index, educational status and a food-frequency questionnaire (FFQ) were obtained with the use of interviewer administered questionnaire. Details on the reproducibility and validity of the FFQ and our data collection procedures were presented in our previous reports^31–33^.

Body mass index (BMI) was calculated as the weight in kilogram divided by height in meter squared (kg/m^2^). Waist and hip circumference were measured in centimeters using constant tension tapes (Seca^®^, Germany). The waist to height ratio (WHtR) was calculated as the waist circumference divided by height in centimeters. Fasting plasma glucose concentrations was measured using the glucose oxidase method, and determined within 1 h of collection. Standard operating procedures were followed to blood sample collection, anthropometric, blood pressure measurements^35^.

### Statistical methods

The data were double entered to Epidata 3.1 and exported to SPSS version 25 ([SPSS Inc., Chicago, Illinois] for analysis. Categorical variables were described as percentages and compared using Pearson chi-square test. Continuous variables were reported as mean and standard deviation (± SD) and compared using independent t-test. Household wealth index score was generated from the data using principal component analysis and grouped into wealth tertile. Based on the participants’ fruits and vegetable consumption, two major dietary patterns were identified as healthy and unhealthy dietary pattern using K-mean cluster analysis.

Linear regression analysis was employed to evaluate the association of prenatal famine exposure with level of fasting blood glucose. The estimated beta-coefficients with their 95% CI were reported. Five sets of regression models were developed to account for the effect of each covariate on the outcome variable. Model 1 was unadjusted, and included the outcome and main exposure variable (prenatal famine exposure variable). Model 2 was built on model 1 and further adjusted for sex. Model 3 was adjusted as model 2 and socio demographic variables including age, residence, educational status, wealth index and family size. Model 4 was adjusted as for model 3 and dietary pattern, physical activity level and family history of diabetes, alcohol and cigarette smoking). Finally, Model 5 was adjusted as Model 4 and Waist to height ratio and BMI. A two-sided P value < 0.05 was found to be statistically important.

### Ethics approval and consent to participate

Permission to conduct the study was obtained from the Institutional Review Board of Jimma University, Institute of Health Sciences, Ethiopia (reference no. JHRPGD/660/2019). Detailed description of the study was given to community leaders and households with the aim of sensitizing and mobilizing the local population. Informed verbal and written consent were taken from each participating household heads.

## Results

### Background characteristics of study participants

A total of 441 (222 exposed and 219 non-exposed) participants were involved in this study with a response rate of 96.7%.The mean (± SD) age of the prenatal famine exposed and non-exposed participants was 35.14(± 0.86) and 31.25 (± 0.65) years respectively, with no significance difference across wealth index (*P* = 0.64), Educational status (*P* = 0.30), dietary pattern (*P* = 0.22), currently smoking (*P* = 0.20), current alcohol drinking (*P* = 0.29) , BMI (*P* = 0.57), Weight to height ratio (*P* = 0.39) and family history of DM (*P* = 0.16) (Table 1).

**Table 1:** Background characteristics of Ethiopian great famine exposed and non-exposed groups in North Wollo Zone, Raya Kobo district, Northeast Ethiopia, 2019.

Variables	Prenatal exposed (n = 222)	Non-exposed (n = 219)	*P value*
Age, (years), mean ± SD	35.14 ± 0.86	31.25 ± 0.65	< 0.001*
Sex, n (%)			
Female	137 (62.6)	118 (53.2)	0.03*
Male	82 (37. 4)	104 (46.8)	
Residence, n (%)			
Urban	41 (19.6 )	43 (18.5)	0.42*
Rural	181 (80.4)	176 (81.5)	
Educational status, n (%)			
No Formal education	66 (29.7)	71 (32.4)	0.30
Formal education	156 (70.3)	156 (67.6)	
Household wealth index, n (%)			
Low	74 (33.3)	45 (20.5)	0.42
Medium	110 (49.5)	88 (40.2)	
High	38 (17.2)	86(39.3)	
Marital status, n (%)			
Single	33 (14.6)	59 (26. 9)	0.04*
Married	145 (64.0)	145 (63.5)	
Divorced/widowed	44 (19.4)	21 (9.6)	
Physical activity level, n (%)			
Low	4 (1.8%)	8 (3.7%)	0.28
Moderate	39 (17.6%)	24 (10.9%)	
High	179 (80.6%)	193 (85.4%)	
Dietary pattern, n (%)			
Healthy	70 (31.5%)	66 (30.1%)	0.41
Unhealthy	152 (68.5%)	153 (69.9%)	
Currently smoking, n (%)			
Yes	37 (16.7%)	35 (16.0%)	0.40
No	185 (83.3%)	184 (84.0%)	
Currently drinking alcohol, n (%)			
Yes	76 (34.2%)	85 (38.8%)	0.18
No	146 (65.8%)	134 (61.2%)	
BMI (kg/m^2^) mean ± SD	22.98 (3.7)	22.68(4.0)	0.57
Fasting blood sugar (mg/dl)	86.40 ± 20.40	82.49 ± 16.85	0.00*
Weight to height ratio mean ± SD	50.9(6.8)	50.3(7.0)	0.39*
Family history of DM			
Yes	3 (1.4%)	7(3.6%)	0.16
No	219 (98.6%)	217 (96.4%)	

*P* value—represents Independent Samples t-tests for continuous variables or χ2-test for categorical variables, *Statistical significance.

### Association of prenatal famine exposure and fasting blood glucose level in adults

In linear regression, unadjusted model (Model 1), the fasting blood glucose level was increased by 4.13 (β = 4.13; 95% CI 0.41, 7.42) points in prenatal famine exposed groups, compared with non-exposed. Similarly, the positive association of prenatal famine exposure and fasting blood glucose level was maintained after adjusted for sex (Model 2) (β = . 4.08 95% CI 0.056, 7.50). Further adjusted for socio demographic variables including age, residence, educational status, wealth index and family size (Model 3) resulted in 4.10 (β = . 4.10 95% CI 0.45, 7.56) points increases in fasting blood glucose level. In model 4 the positive association of prenatal famine exposure and fasting blood glucose was maintained after adjusted for dietary pattern, physical activity level and family history of diabetes, alcohol and cigarette smoking (β = 3.90, 95% CI 039, 7.52). In the full adjusted (Model 5) prenatal exposure to famine resulted in 3.78 (β = 3.78, 95% CI 0.22, 7.34) increases in fasting blood glucose level after adjusted for BMI and waist to height ratio (Table 2).

**Table 2:** Association of prenatal famine exposure and fasting plasma glucose among adults, a linear regression analysis, Wollo, Ethiopia.

Models	β (95.0% CI)	Covariates
Model 1	4.13 (0.41, 7.42*)	Unadjusted
Model 2	4.08 (0.056, 7.50*)	Model 1 adjusted Sex
Model 3	4.10 (0.45, 7.56*)	Sex, age, residence, educational status, wealth index, family size
Model 4	3.90 (0.039, 7.52*)	Sex, age, residence, educational status, wealth index, dietary pattern, physical activity level, family history of DM, current alcohol drinking, current smocking
Model 5 (fully adjusted model)	3.78 (0.22, 7.34)	Age, sex, residence, educational status, wealth index, dietary pattern, physical activity level, family history of DM BMI, Hait to Height Tatio

*Significant at *P* < 0.01, **significant at *P* < 0.05, fasting blood sugar, BMI; body mass index, BP; blood pressure.

All β-coefficients (95% CI) were from multiple linear regression analysis, and related to the non-exposed groups.

## Discussion

The present historical cohort study investigated the impact of prenatal exposure to famine on adulthood fasting plasma glucose level. The finding indicated that people who had been exposed to famine in prenatal period had significantly higher glucose concentrations as compared to non-exposed groups. This finding is in line with previously reported famine studies^19,28^. However, it also contrasted the findings of other studies which reported null to inverse association of early childhood famine exposure and adulthood blood glucose parameters^14–18^. The possible reason for these inconsistent observations could be explained in terms of the heterogeneity of study’s methodology, the exposure characteristics such as duration of famine, severity, age during the exposure and the observation, and other covariates. For example, the Chinese Great Famine lasted much longer and had greater severity for which the food supplies were not sufficiently recovered until 20 years later in the 1980s^36^. In contrast, the Dutch famine was relatively for shorter period^10^, while the Ethiopian famine was for two years^11,12^.

Corroborating our finding, many epidemiological surveys and studies involving children with low birthweight reported a link between early childhood nutrition deprivation and increased risk of diabetes^19–28^. Similarly, proxy to famine exposure, survivors of severe childhood malnutrition were also found to have reduced insulin sensitivity, or glucose intolerance^37–40^. Of particular interest, Boulé et al. (2003), reported that participants with early life malnutrition would display a more pronounced deterioration of insulin sensitivity in association with a gain in abdominal fat^37^. Interestingly, studies which employed animal models of starvation also suggest lower expression of specific genes related to insulin signaling and nutrient sensing^41^, growth and development of pancreatic beta cell^42^ and skeletal muscle^43^. Furthermore, genetic studies also reported the possible link of famine associated epigenetic changes altering the expression of genes involved in metabolic pathway and pancreatic beta cell functioning^44,45^.

Our study finding possesses paramount importance as it reflects metabolic conditions of participants in the early adulthood, in contrast to most famine studies which reported impact of childhood famine on older adults^13–29^. Furthermore, none of the studied covariates in the present study nullify or modified the positive association between prenatal famine and fasting blood glucose level, affirming the independent association of the exposure. This observed phenomenon may imply that, in early adulthood, the impact of most classic covariates like family history diabetes, alcohol intake, cigarette smoking and sedentary behavior over blood glucose control may not be as strong as prenatal famine. Nonetheless, in the present cohort, weight to height ratio was the only predictor associated with raised fasting blood glucose level. This finding is decisive considering similar reports indicating elevated risk of diabetes in the prenatal famine exposed cohorts categorized as overweight/obese^17,24,25^.

The implication of our study should be interpreted in the context of the prevalent under nutrition in low income countries, which could have close or similar consequences. Nevertheless, there remained certain limitation in our study, which could encumber the interpretation of the findings; first, it should be noted that survivors are biologically unique, hence it could likely introduce selection bias. Second; severity of the famine exposure at the individual level was not considered. Thirdly, early childhood experiences, parent–child bond and maternal factors during early life was not accounted. Lastly, even though we counted age in regression, it should be noted that the control groups were younger.

In conclusion, there existed a positive association of prenatal famine exposure and blood glucose level during adulthood. In such population, the need for effective overweight/obesity prevention program to override the negative effect of early famine exposure on blood glucose control is critical undertaking.

## Supplementary Information

Supplementary Information.

## Data Availability

The data supporting the conclusions of this article is included within the article (and its Additional file).

## Abbreviations

CI: Confidence interval
BMI: Body mass index
COR: Crude odds ratio
AOR: Adjusted odds ratio
PCA: Principal component analysis
FFQ: Food frequency questionnaire (FFQ)
SD: Standard deviation
