# Supplementary material for: Impact of prenatal famine exposure on adulthood fasting blood glucose level
Source: Sci Rep. 2022 Apr 13;12:6198. doi: 10.1038/s41598-022-10120-3 (PMC9008050; doi:10.1038/s41598-022-10120-3)
Supplement: Supplementary file 1 — Supplementary Information. [file 41598_2022_10120_MOESM1_ESM.docx]

Registration was carried out to prepared sampling frame

Total subjects (n **456**)

Proportional allocation to selected kebeles then participants are selected by simple random sampling techniques

Exclusion

Missing values of laboratory data (n **15**)

Participants included in the study (n **441**)

1. Non- exposed groups (n **219**)
2. Prenatal-exposed groups (n **222**)

Supp. **Figure** **1**. Flow diagram representing sample recruitment.
